# Phosphoinositide‐ and Collybistin‐Dependent Synaptic Clustering of Gephyrin

**DOI:** 10.1111/jnc.70169

**Published:** 2025-08-08

**Authors:** Nele Burdina, Filip Liebsch, Arthur Macha, Joaquín Lucas Ortuño Gil, Pia Frommelt, Irina Rais, Fabian Basler, Simon Pöpsel, Guenter Schwarz

**Affiliations:** ^1^ Department of Chemistry and Biochemistry, Institute of Biochemistry University of Cologne Cologne Germany; ^2^ Center for Molecular Medicine Cologne (CMMC), Faculty of Medicine and University Hospital University of Cologne Cologne Germany; ^3^ Cologne Excellence Cluster on Cellular Stress Responses in Aging‐Associated Diseases (CECAD) University of Cologne Cologne Germany

**Keywords:** collybistin, GABA receptors, gephyrin, inhibitory synapse, phosphoinositides, synaptic clustering

## Abstract

Gephyrin is the main scaffolding protein at inhibitory synapses, clustering glycine and GABA_A_ receptors. At specific GABAergic synapses, the nucleotide exchange factor collybistin recruits gephyrin to the postsynaptic membrane via interaction with phosphoinositides. However, the molecular mechanisms underlying the formation, maintenance, and regulation of collybistin‐dependent gephyrin clusters remain poorly understood. This study sheds light on the molecular mechanism of gephyrin cluster formation on the basis of gephyrin self‐oligomerization induced by collybistin, leading to the formation of a high‐molecular weight (> 5 MDa) gephyrin‐collybistin complex, which is regulated in two ways: First, plasma‐membrane phosphoinositides promote complex formation, demonstrating their critical role in membrane targeting and stabilization of gephyrin‐collybistin clusters at postsynaptic sites. Second, gephyrin phosphorylation at Ser325 abolishes complex formation with collybistin, thus impairing collybistin‐dependent gephyrin clustering at GABAergic synapses. Collectively, our data demonstrate a molecular mechanism for synaptic clustering of gephyrin, which involves collybistin‐ and phosphoinositide‐dependent formation of high‐molecular weight gephyrin oligomers.

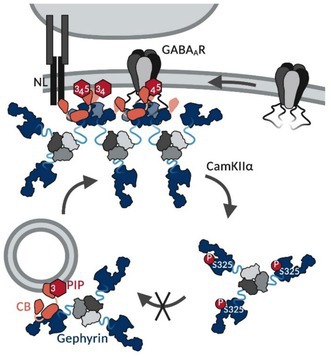

## Graphical abstract

Gephyrin is the main scaffolding protein at inhibitory synapses, clustering glycine and GABA_A_ receptors. At specific GABAergic synapses, collybistin recruits gephyrin to the postsynaptic membrane via interaction with phosphoinositides. Here, we show that collybistin not only mediates membrane targeting but also induces gephyrin self‐oligomerization into a high‐molecular weight (> 5 MDa) complex. This process is regulated by two mechanisms: Membrane‐resident phosphoinositides promote high‐molecular weight gephyrin‐collybistin complex formation at postsynaptic sites, whereas gephyrin phosphorylation at Ser325 disrupts collybistin binding and clustering at GABAergic synapses. Collectively, our results demonstrate a molecular mechanism for synaptic clustering of gephyrin, involving collybistin‐ and phosphoinositide‐dependent formation of high‐molecular weight gephyrin oligomers.

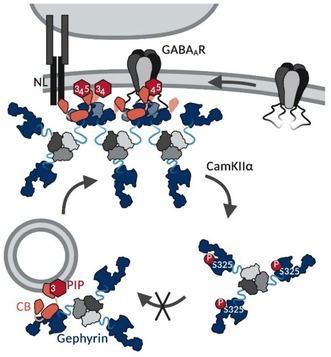

**Abbreviations**
AU: absorption unit
CB: collybistin
CB2_SH3‐_
: collybistin isoform 2 lacking the SH3 domain
CB2_SH3+_
: collybistin isoform 2 containing the SH3 domain
ddGeph: dimerization‐deficient gephyrin variant
DDM: dodecyl‐β‐d‐maltosid
DH domain: Dbl homology‐domain
Folch: bovine brain lipid extract Folch fraction I
GABA_A_R: γ‐aminobutyric acid type A receptor
Geph: gephyrin
GephE: gephyrin E‐domain
GephHO: gephyrin high oligomers
GephT: gephyrin trimers
GlyR β‐ICD: GlyR β‐loop, residues 378–426
GlyR: glycine receptor
HEK cells: human embryonic kidney 293 cells
HEK GPHN−/−: gephyrin knock‐out HEK cells
ICD: intracellular cytosolic domain
ITC: isothermal titration calorimetry
LS: lumazine synthase
LS‐αlCD: lumazine synthase with included GlyR‐α1ICD
LS‐αβlCD: co‐expressed Lsαl and Lsβl
LS‐βlCD: lumazine synthase with included GlyR‐βICD
Moco: molybdenum cofactor
MPT: molybdopterin
MW: molecular weight
NL: neuroligin
P1–3: peak 1–3
PBS: phosphate‐buffered saline
PEI: polyethylenimine
PH domain: pleckstrin homology domain
PI(3)P: phosphatidylinositol‐3‐monophosphate
PI(3,4)P2: phosphatidylinositol‐3,4‐bisphosphate
PI(3,4,5)P2: phosphatidylinositol‐3,4,5‐trisphosphate
PI(3,5)P2: phosphatidylinositol‐3,5‐bisphosphate
PI(4)P: phosphatidylinositol‐4‐phosphate
PI(4,5)P2: phosphatidylinositol‐4,5‐bisphosphate
PIP: phosphoinositide
POPC: 1‐palmitoyl‐2‐oleoyl‐sn‐glycero‐3‐phosphocholine
PTMs: post translational modifications
rAAVs: recombinant adeno‐associated virus
RRID: Research Resource Identifier (see scicrunch.org)
SEC: size exclusion chromatography
SH3 domain: regulatory src homology 3‐domain
vGAT: vesicular GABA transporter
WT: wild‐type

## 1 Introduction

Efficient synaptic transmission requires precise accumulation of neurotransmitter receptors at the postsynaptic membrane. Scaffolding proteins provide structural support for various postsynaptic components, including neurotransmitter receptors, adhesion molecules, and other functional elements (Sheng and Kim 2011). At inhibitory postsynapses, gephyrin serves as the major scaffolding protein, clustering glycine receptors (GlyR) and specific subtypes of γ‐aminobutyric acid type A receptors (GABA_A_R) (Fritschy et al. 2008). In addition to gephyrin's role in neuronal scaffolding, it catalyzes the last two steps of the molybdenum cofactor (Moco) biosynthesis (Feng et al. 1998; Stallmeyer et al. 1999). Gephyrin is essential for postnatal survival, as gephyrin‐deficient mice die within the first postnatal day because of a lack of synaptically localized inhibitory receptors and impaired molybdoenzyme activity (Feng et al. 1998). In humans, gephyrin dysfunction has a severe impact on neurotransmission and has been associated with various brain disorders, including epilepsy, Dravet‐like syndrome, epileptic encephalopathy, schizophrenia, and autism spectrum disorder (Dejanovic et al. 2014, 2015; Lionel et al. 2013; Macha, Liebsch, et al. 2022).

Gephyrin is composed of an N‐terminal G‐, a central C‐, and a C‐terminal E‐domain. The isolated G‐domain and E‐domain form trimers and dimers, respectively (Kim et al. 2006; Schwarz et al. 2001; Sola et al. 2001). Gephyrin E‐domain dimers provide binding sites for the receptors and, together with G‐domain trimers, contribute to the formation of higher order oligomers within the postsynaptic gephyrin scaffold (Herweg and Schwarz 2012; Pennacchietti et al. 2017; Specht et al. 2013). Interestingly, previous in vitro studies using recombinant full‐length gephyrin found trimeric gephyrin with monomeric E‐domains because of an inhibitory role of the central C‐domain (Bedet et al. 2006; Sander et al. 2013). Therefore, the molecular mechanisms triggering E‐domain dimerization at postsynaptic sites remain elusive and are thought to involve dynamic regulation via post‐translational modifications (PTMs) and interacting proteins, relieving the inhibitory effect of the C‐domain (Bedet et al. 2006; Sander et al. 2013).

**FIGURE 1:**
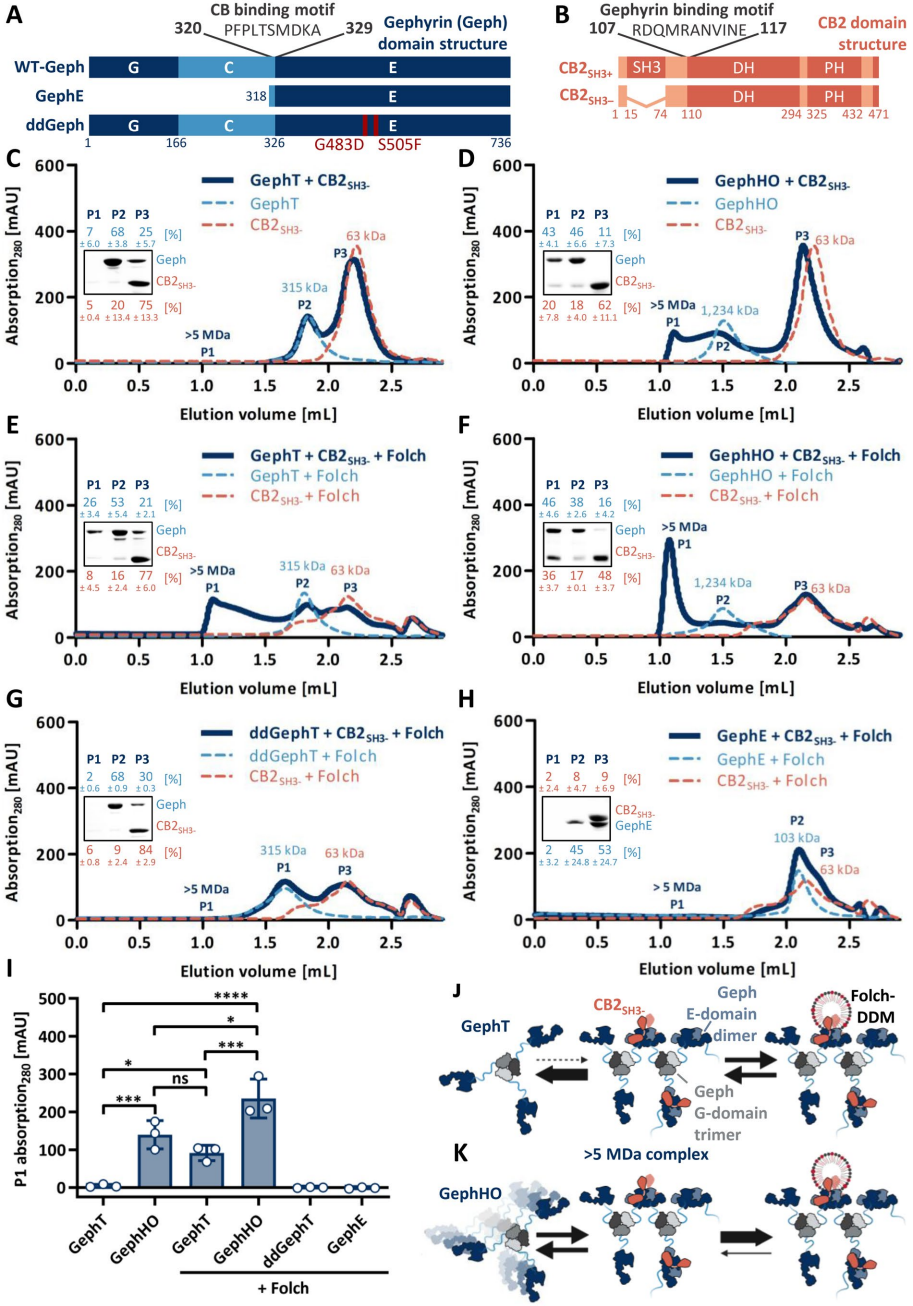
High‐molecular weight gephyrin‐CB2_SH3‐_ complex formation is dependent on gephyrin self‐oligomerization and promoted by Folch lipids. (A) Domain structure of the used gephyrin (Geph) variants with the CB binding motif highlighted (Harvey et al. 2004). (B) Domain structure of CB2 with and without SH3 domain. The gephyrin binding motif is highlighted (Grosskreutz et al. 2001; Tyagarajan, Ghosh, Harvey, and Fritschy 2011). (C–H) Representative SEC elution profiles of gephyrin variants mixed with CB2_SH3‐_ at equimolar ratios (dark blue line), alone or in the presence of Folch. Single gephyrin (dashed line, light blue) and CB2_SH3‐_ (dashed line, orange), with or without Folch, serve as a reference within each graph. The MWs of the single proteins as well as the formed complexes, determined according to the standard protein calibration curve, are indicated. Insets depict SDS‐PAGE analysis of peak 1 (P1), peak 2 (P2), and peak 3 (P3) of the respective gephyrin‐CB2_SH3‐_ interaction runs. Numbers above and below the representative SDS‐PAGE image represent the mean relative band intensity ± SD [%] of gephyrin (light blue) and CB2_SH3‐_ (orange), respectively, between P1, P2, and P3 (*n* = 3 from three independently purified protein batches). (I) Quantification of P1 absorption at 280 nm expressed as mean ± SD (*n* = 3 from three independently purified protein batches). ddGephT and GephE are shown as a control depicting an absent gephyrin‐CB2_SH3‐_ complex formation. The different oligomeric states of full‐length WT‐Geph were analyzed by one‐way ANOVA (F(5,12) = 37.89, *p* < 0.0001, Bonferroni post hoc test: GephT vs. GephHO *p* = 0.0008 (***); GephT vs. GephT+Folch *p* = 0.0299 (*); GephT vs. GephHO+Folch *p* < 0.0001 (****); GephHO vs. GephT+Folch *p* = 0.7542 (ns); GephHO vs. GephHO+Folch *p* = 0.0151 (*); GephT+Folch vs. GephHO + Folch *p* = 0.0004 (***)). (J, K) Proposed model for the formation of the > 5 MDa gephyrin‐CB2_SH3‐_ complex via gephyrin E‐domain dimerization induced by CB2_SH3‐_ and stabilized by Folch lipids comparing (J) GephT and (K) GephHO (created with BioRender.com).

At GABAergic synapses, the brain‐specific GTP/GDP exchange factor collybistin (CB) is a critical interactor, binding to the domain boundary of the gephyrin C‐ and E‐domain (Figure 1A) (Harvey et al. 2004). Experiments with CB‐deficient mice revealed that CB is essential for the formation and maintenance of α2‐/γ2‐subunit‐containing GABA_A_R clusters that recruit gephyrin in various brain regions, including the hippocampus, cerebellum, and basolateral amygdala (Papadopoulos et al. 2007, 2008). There are several CB splice variants (CB1‐CB3), which all contain a tandem Dbl homology (DH) and a pleckstrin homology (PH) domain but differ in their N‐ and C‐termini as well as the presence of an N‐terminal regulatory src homology 3 (SH3) domain (Harvey et al. 2004). The PH domain of CB interacts with phosphoinositides (PIPs) and was shown to mediate gephyrin transport towards synaptic membranes via interaction with phosphatidylinositol‐3‐monophosphate (PI(3)P) located at early/sorting endosomes (Kalscheuer et al. 2009; Papadopoulos et al. 2017). SH3 domain‐containing CB isoforms adopt a closed, autoinhibited conformation that hinders PIP binding and prevents gephyrin recruitment to the membrane (Soykan et al. 2014; Xiang et al. 2006). Several interaction partners have been shown to relieve the autoinhibited conformation of CB by binding to either its SH3 or PH domain. The SH3 domain of CB binds synaptic proteins such as the GABA_A_R α2‐subunit (Saiepour et al. 2010) as well as the neuroligins NL2 and NL4 (Hoon et al. 2011; Poulopoulos et al. 2009; Soykan et al. 2014), whereas the PH domain interacts with the GTPase TC10 (Kilisch et al. 2020; Mayer et al. 2013). These interactions trigger a conformational switch within CB toward an open conformation that enables phosphoinositide binding. Notably, NL2 and prolonged TC10 interaction have been shown to induce a phospholipid affinity switch of CB towards plasma‐membrane‐resident PIPs, such as PI(3,4)P_2_, PI(4,5)P_2_, and PI(3,4,5)P_3_, facilitating synaptic clustering of the gephyrin‐CB complex (Kilisch et al. 2020; Schäfer et al. 2020). Impairments in CB‐dependent synaptic gephyrin clustering lead to severe neurological dysfunctions such as epilepsy, anxiety, aggression, and intellectual disabilities (Chiou et al. 2019; Kalscheuer et al. 2009). Despite the fundamental importance of CB‐induced gephyrin cluster formation at GABAergic synapses, the molecular basis of formation, maintenance, and regulation of gephyrin‐CB clusters remains poorly understood.

Here, we characterized the molecular mechanism of gephyrin‐CB complex formation by studying the oligomerization of gephyrin upon CB binding, as well as the modulation of this interaction by PIPs and PTMs. We revealed that CB binding induces gephyrin self‐oligomerization, leading to the formation of a high‐molecular weight (> 5 MDa) gephyrin‐CB complex, which was dependent on gephyrin E‐domain dimerization. Our data showed that gephyrin‐CB complex formation can be modulated in two directions: plasma‐membrane‐resident PIPs present at the postsynaptic membrane stabilize the complex and gephyrin phosphorylation at Ser325 inhibits the complex formation.

## 2 Materials and Methods

### 2.1 Expression Constructs

His_6_‐tagged rat gephyrin P1 (Gehling et al. 2024) and rat gephyrin E‐domain (GephE) (Belaidi and Schwarz 2013) constructs in pQE80L were described previously and were used for recombinant *E. coli* expression and purification. The amino acid exchanges for the generation of ddGeph, ddGephE (G483D and S505F), and the phosphomimicking gephyrin variants (T324D‐Geph and S325D‐Geph) were generated by site‐directed mutagenesis.

For gephyrin expression in HEK GPHN^**−/−**^ cells, the construct GFP‐Geph in pEGFP‐C2 (Clonetech) was cloned via Gibson cloning using gephyrin P1 in pQE80L as a template. GFP‐tagged S325D‐ and S325A‐Geph variants were generated by site‐directed mutagenesis. The construct pAAV‐hSyn‐fDIO‐mScarlet‐Gphn_P1 used for virus production for neuronal expression of gephyrin was described previously (Addgene plasmid # 194972; http://n2t.net/addgene:194972; RRID:Addgene_194 972). S325D‐Geph in pQE80L was used as a template to generate pAAV‐hSyn‐fDIO‐mScarlet‐Geph‐S325D via Gibson assembly.

Rat CB2_SH3‐_ in mCherry‐C3 (Dejanovic et al. 2015) was subcloned into pQE80L (Qiagen) via PCR using BamHI and SalI restriction sites for recombinant *E. coli* expression and purification. For expression in HEK GPHN^**−/−**^ cells, an N‐terminal myc‐tag was added via overlap extension PCR, and myc‐CB2_SH3‐_ was subcloned into pcDNA3.1 Myc/His B (Invitrogen) using Gibson cloning.

The *E. coli* expression constructs of LS‐αβICD in pETDuet1 (Macha, Grünewald, et al. 2022) and Intein‐tagged GlyR β‐loop in pTYB2 (Schrader et al. 2004) were described previously.

### 2.2 Protein Purification

Recombinant His_6_‐tagged full‐length gephyrin variants (WT‐Geph, ddGeph, T324D‐Geph, S325D‐Geph) were expressed in *E. coli* BL21 Rosetta cells for 16 h at 18°C, whereas His_6_‐tagged gephyrin E‐domain variants (WT‐GephE and ddGephE) were expressed for 16 h at 30°C. Cells were harvested by centrifugation (5000 *g*, 10 min). The cell pellet was resuspended in lysis buffer (100 mM Tris/HCl pH 7.5, 250 mM NaCl) supplemented with 0.05% (v/v) Tween‐20, lysozyme (Sigma), protease inhibitors (cOmplete EDTA‐free, Roche), and DNAse I (Roche) and stored at −20°C until further use. All full‐length gephyrin and gephyrin E‐domain variants were affinity‐purified using nickel‐nitrilotriacetic acid resin (Ni‐NTA, Cube Biotech) followed by preparative size exclusion chromatography (SEC) as described in the following: Cells were lysed by two cycles of sonication (3 min, 30 s pulse, 30 s pause, 40% amplitude) and pressure lysis (1000–1500 bar, EmulsiFlex high‐pressure homogenizer, Avastin). After pelleting cell debris (50 000 *g*, 1 h), the supernatant was incubated with Ni‐NTA beads. Unbound proteins were removed by several washing steps using lysis buffer supplemented with 10 mM imidazole, followed by lysis buffer supplemented with 20 mM imidazole. The protein was eluted in elution buffer (100 mM Tris/HCl pH 7.5, 250 mM NaCl, 400 mM imidazole). To further purify the protein and to separate the specific oligomeric states of gephyrin, preparative SEC was performed using a Superdex 200 16/60 column (120 mL column volume, GE Healthcare) equilibrated with storage buffer (25 mM Tris/HCl pH 7.5, 250 mM NaCl, 5 mM β‐mercaptoethanol, 5% (v/v) glycerol).

Recombinant His_6_‐tagged CB2_SH3‐_ was expressed in *E. coli* BL21 Rosetta cells for 16 h at 18°C. Cell harvesting, NiNTA affinity purification, as well as preparative SEC were carried out as described for gephyrin, except modified buffer compositions (Lysis buffer: 100 mM Hepes pH 8.0, 500 mM NaCl, 10% (v/v) glycerol, 1% (w/v) CHAPS, 1 mM β‐mercaptoethanol; Elution buffer: 100 mM Hepes pH 8.0, 500 mM NaCl, 10% (v/v) glycerol, 50 mM arginine, 50 mM glutamate, 300 mM imidazole, 1 mM β‐mercaptoethanol; Storage buffer: 25 mM Tris/HCl pH 7.5, 250 mM NaCl, 10% glycerol, 10 mM EDTA, 5 mM β‐mercaptoethanol).

Recombinant Intein‐tagged GlyR β‐loop was expressed in *E. coli* ER2566 for 16 h at 18°C, and the affinity purification was carried out using the IMPACT protein purification system (New England Biolabs) according to a previously described protocol (Schrader et al. 2004). After affinity purification, the GlyR β‐loop was further purified by preparative SEC using a Superdex 200 16/60 column (120 mL column volume, GE Healthcare) equilibrated with storage buffer.

Recombinant LS‐αβICD, carrying a His_6_‐tag at the LS‐αICD subunit, was expressed and purified using Ni‐NTA affinity purification followed by preparative SEC using storage buffer as previously described (Macha, Grünewald, et al. 2022).

All proteins were flash frozen in liquid nitrogen and stored at −80°C until further use.

### 2.3 Detergents and Lipids

Dodecyl‐β‐D‐maltosid (DDM) was purchased from GLYCON Biochemicals GmbH, 1‐palmitoyl‐2‐oleoyl‐sn‐glycero‐3‐phosphocholine (POPC), Folch lipids (Type I, Folch Fraction I) from Sigma, and PIPs (PI(3)P, PI(4)P, PI(3,4)P_2_, PI(3,5)P_2_, PI(4,5)P_2_, PI(3,4,5)P_3_, C_16_ derivatives) from MoBiTec GmbH.

Stock solutions of POPC and Folch were prepared in chloroform, whereas the PIPs were dissolved in a mixture of chloroform, methanol, and water according to the manufacturer's instructions. POPC and PIP stock solutions were mixed and added to chloroform to obtain the desired lipid molar ratios of 10 mol% of the different phosphoinositides in POPC. The organic solvents were evaporated with a gentle argon stream. Lipid films were further dried under vacuum overnight. The lipid film was dissolved in DDM buffer (25 mM Tris/HCl pH 7.5, 250 mM NaCl, 1% (w/v) DDM) by three cycles of vortexing (1 min), followed by sonication (10 min, SONOREX RK100H, Bandelin) to generate the desired lipid‐DDM micelles (PIP‐POPC‐DDM micelles; Folch‐DDM micelles).

### 2.4 SEC Interaction Studies

The complex formation between the different gephyrin variants and CB2_SH3‐_ as well as the self‐oligomerization of gephyrin was analyzed by analytical SEC using a Superose 6 increase 5/10 column (3 mL, GE Healthcare). Therefore, the respective proteins were mixed at an equimolar ratio using 1.5 nmol each. For the conditions using lipids, additional 1.5 nmol Folch lipids in DDM micelles or 1.5 nmol of the respective PIP‐lipids in POPC‐DDM micelles were added to the mixture. The samples were adjusted to a volume of 90 μL using storage buffer and afterward incubated for 30 min on ice. After centrifugation (17 000 *g*, 10 min, 4°C), the samples were applied to the column and proteins were separated at 4°C and a flow rate of 0.2 mL/min in SEC buffer (25 mM Tris/HCl pH 7.5, 150 mM NaCl, 5 mM β‐mercaptoethanol). For SEC runs in the presence of lipid‐DDM micelles, the SEC buffer was supplemented with 0.01% DDM. Elution was monitored by monitoring absorbance at 280 nm. MWs were determined by comparison to the elution of standard proteins (Figure S2A). 100 μL fractions was collected and the desired peak fractions were further subjected to Coomassie stained SDS‐PAGE analysis.

### 2.5 ITC Interaction Studies

Isothermal titration calorimetry (ITC) experiments were performed by titrating the GlyR β‐loop (300 μM) into the respective gephyrin variants (30 μM) using a MicroCal Auto‐ITC200 (Malvern). The used proteins were within the same batch of storage buffer. The ITC measurements were carried out at 37°C using an injection volume of 1.25–1.5 μL, a spacing of 120 s between injections, and an initial delay of 60 s. The reference power was set to 5 μCal/s, and the stirring speed to 750 rpm. Parameters and curves were fitted and calculated by the MicroCal Analysis software and Origin7.

### 2.6 In Vitro Moco Assay

The in vitro Moco synthesis assay was performed as previously described (Belaidi and Schwarz 2013). For the analysis of Moco production of gephyrin in complex with CB2_SH3‐_, both proteins were mixed at an equimolar ratio at a concentration of 25 μM either in the absence or presence of 25 μM Folch‐lipids in DDM micelles. 300 pmol of the complex or the respective gephyrin variant alone was used for the assay. Samples without gephyrin (‐Geph) or without molybdenum (‐Mo) were used as control and showed Moco produced either chemically or by trace amounts of molybdenum in the buffers.

### 2.7 SDS‐PAGE, Coomassie, and Western Blot

Samples were supplemented with 1× sample buffer (5×: 250 mM Tris/HCl pH 6.8, 30% glycerol, 0.1% Bromophenol blue, 10% SDS, 5% β‐mercaptoethanol) and incubated for 5 min at 95°C. Protein separation was performed with 12% SDS acrylamide gels, followed by Coomassie staining (30% EtOH, 10% acetic acid, 0.25% Coomassie brilliant blue R250) or immunoblotting using standard protocols with a chemiluminescence and an ECL system. The following antibodies were used and diluted in Tris‐buffered saline/0.5% Tween containing 1% dry milk: anti‐gephyrin E‐domain (3B11, 1:10, self‐made, RRID: AB_887719); anti‐GAPDH (1:1000, G9545, Sigma), anti‐mouse HRP‐coupled (1:10 000, AP181P, Sigma); anti‐rabbit HRP‐coupled (1:10 000, AP187P, Sigma). Image acquisition of Coomassie stained gels and immunoblot detection were performed with a ChemiDocTM Imaging System (BioRad). Band intensities were quantified using Image Lab 6.1 (BioRad).

### 2.8 Generation of HEK GPHN ^−/−^ Cells

Human embryonic kidney (HEK293, not authenticated) cells were cultured in Dulbecco's modified Eagle's medium supplemented with 10% FCS and 2 mM l‐glutamine at 37°C and 5% CO_2_. HEK293 cells are not listed as a commonly misidentified cell line by the International Cell Line Authentication Committee (ICLAC version 13, released 26 April 2024). Gephyrin‐deficient HEK293 cells were generated using the double‐nickase CRISPR/cas9 approach described previously (Ran et al. 2013). Two pX335 constructs (Addgene #42335), each encoding one *GPHN*‐specific guide RNA (gRNA 1: TACTAACCACGACCATCAAA; gRNA 2: CTCAGGAATGTCCATTGGCC) were generated. HEK293 cells were transfected daily with both pX335 constructs for 5 days using GeneJuice transfection reagent (Novagen) according to manufacturer's protocol. After 4 days, cells were split 1:2. After 5 days of daily transfection, putative knock‐out candidates were separated from WT cells via limiting dilution. Therefore, cells were detached via trypsin/EDTA treatment, diluted to a concentration of 0.5 cells/100 μL and 100 μL were plated into 96‐well plates. Single colonies were grown to ~70% confluency and transferred onto 12‐well plates. HEK293 GPHN^−/−^ cells were identified via western blot.

### 2.9 HEK GPHN ^−/−^ Transfection

HEK293 GPHN^−/−^ cells were plated onto poly‐l‐lysine coated glass coverslips and transfected using 200 ng of the respective plasmid DNA with polyethylenimine (PEI, Sigma) according to the manufacturer's protocol. 14 h after PEI transfection, cells were fixed using 4% paraformaldehyde in phosphate‐buffered saline (PBS) for 10 min, followed by a PBS washing step. For immunocytochemistry, cells were permeabilized for 10 min with PBS supplemented with 0.2% Triton X‐100. After blocking with 1% BSA in PBS for 5 min, cells were incubated with the primary anti‐myc antibody (9E10) for 2 h. Subsequently, the secondary antibody (Alexa Fluor 647 goat anti‐mouse; 1:500; Invitrogen) was applied for 1 h. Cells were then washed three times with PBS. Coverslips were mounted onto glass slides using a homemade Mowiol/Dabco solution, dried overnight at room temperature, and stored at 4°C.

### 2.10 Virus Preparation

Production of rAAV particles was carried out in HEK293 cells, followed by PEG/NaCl precipitation and chloroform extraction according to a previously described protocol (Kimura et al. 2019). Purity of the rAAVs was assessed using SDS‐PAGE, and titers were determined using Gel green (Biotium) according to a previously described protocol (Xu et al. 2020). The fluorescence was detected at 507 ± 5 nm (excitation) and 528 ± 5 nm (emission) using a plate reader with monochromators (Tecan Spark).

### 2.11 Primary Neuron Transfection, Transduction, Fixation, and Immunocytochemistry

Dissociated primary hippocampal cultures were prepared from C57BL/6NRj embryos (Janvier‐Labs) of either sex (E17.5). Therefore, pregnant dams were killed by cervical dislocation. E17.5 embryos were isolated and decapitated as quickly as possible. For each neuron culture, the hippocampi from all embryos of a single dam (typically 6–8 embryos) were pooled. In total, four cultures were prepared, using hippocampi from four dams and 28 embryos. After dissociation, cells were seeded on poly‐l‐lysine coated coverslips in neurobasal medium supplemented with B‐27, N‐2, and l‐glutamine (*Thermo Fisher Scientific*). After 9 days in vitro (DIV), neurons were transfected with 400 ng plasmid DNA using Lipofectamine 2000 (Life Technologies) according to the manufacturer's protocol. After 10 DIV, neurons were infected with 1 × 10^8^ viral genome copies using rAAV particles diluted in neurobasal medium supplemented with B‐27, N‐2, and l‐glutamine (*Thermo Fisher Scientific*). After 15 DIV, cells were fixed using 4% paraformaldehyde in PBS for 15 min, followed by three washing steps using 50 mM ammonium chloride in PBS. For immunocytochemistry, the cells were blocked/permeabilized for 1 h using 10% goat serum, 1% BSA, 0.2% Triton X‐100 in PBS. Cells were incubated for 1 h with the following antibodies: anti‐vesicular GABA transporter (vGAT) (1:1000, #131003) for inhibitory presynaptic terminals; anti‐GABA_A_R γ2 (1:500, #224004) for postsynaptic GABA_A_Rs and subsequently washed using PBS. The following secondary antibodies were used: goat anti‐rabbit AlexaFluor 488 (1:500, #A‐11034, Thermo Fisher Scientific), and goat anti‐guinea pig AlexaFluor 647 secondary antibodies (1:500, #ab150187, Abcam). The coverslips were mounted onto glass slides using a homemade Mowiol/Dabco solution, dried overnight at room temperature, and stored at 4°C.

### 2.12 Confocal Microscopy and Image Analysis

Images were acquired with stacks of 0.3 μm *z*‐step size, 2048 × 2048 (144.77 × 144.77 μm) using the Leica TCS SP8 LIGHTNING upright confocal microscope with an HC PL APO CS2 63×/1.30 glycerol objective. The microscope was equipped with hybrid detectors (Leica HyD) and diode lasers with 405, 488, 522, and 638 nm. LIGHTNING adaptive deconvolution of the mounting medium “Mowiol” was used, which is capable of theoretical resolutions down to 120 and 200 nm lateral and axial, respectively. Images were segmented and analyzed in an automated fashion using ImageJ/FIJI and an adapted previously described macro (Liebsch et al. 2023).

### 2.13 Subcellular Fractionation of Brain Tissue

Fractionation of cytosolic and membrane associated gephyrin from pig brain tissue of either sex was performed as described in the following. In brief, frozen pig brain tissue was mortared into fine powder and dissolved in 10 tissue volumes of tissue lysis buffer (20 mM Hepes pH 8.0, 150 mM NaCl, 5% glycerol, 5 mM EDTA, 50 mM DTT, 20 mM NEM) supplemented with protease inhibitor (cOmplete, Roche). Cells were lysed mechanically using a Potter with Teflon pestle (1100 rpm, 30 strokes) followed by sonication (2 × 10 s, 30% amplitude). Cell debris and unbroken cells were removed by centrifugation at 4000 *g* (4°C, 10 min) and the supernatant was used for ultracentrifugation (185 000 *g*, 1 h, 4°C, Beckman Type 70.1 Ti rotor). The supernatant was used as the cytosol sample and was used directly for blue native PAGE sample preparation. The pellet represents the membrane sample and was dissolved in tissue lysis buffer supplemented with 1% (w/v) DDM and mechanically disrupted using a glass potter (50×). After an incubation time of 1 h (4°C, head over tail), solubilized membranes were centrifuged (185 000 *g*, 30 min, 4°C, Beckman Type 70.1 Ti rotori) and the supernatant was used for blue native PAGE sample preparation.

### 2.14 Blue Native PAGE Analysis

Samples for blue native PAGE analysis were prepared by 1:3 dilution using H_2_O and the addition of 10× blue native loading buffer (312 mM imidazole, 500 mM 6‐aminohexanoic acid, 5% CBB G250, 6.25 mM EDTA, 40% glycerol). Samples were applied to 4%–16% native PAGE (TM Novex Bis‐Tris Gel) and protein separation was performed according to the manufacturer's protocol. Afterward, gels were subjected to western blotting.

### 2.15 Mass Photometry Measurements

Mass photometry measurements were conducted using a Refeyn TwoMP mass photometer (Refeyn Ltd.). Standard microscopy glass slides were cleaned with MilliQ water and isopropanol. Residual lint was removed by a stream of compressed air, and 3 × 2‐well silicon sample well cassettes were attached to the clean glass slides. The detector was cleaned with isopropanol, and the coverslips were mounted onto the detector using one drop of immersion oil. For each mass photometry measurement, the protein samples were pre‐diluted to approximately 100 nM using dilution buffer (25 mM Tris/HCl pH 7.5, 150 mM NaCl, 5 mM β‐mercaptoethanol, 1% (v/v) glycerol). 19 μL of the dilution buffer was placed into one silicon well, and the focal plane was automatically estimated via the droplet dilution feature in the software AcquireMP (Refeyn Ltd.). 1 μL of the diluted sample was added to the measuring chamber, resulting in a final concentration of 5 nM. The measurement was conducted for 60 s, after which the contrast histograms were analyzed in the software DiscoverMP (Refeyn Ltd.) using a previously acquired mass calibration with bovine serum albumin and human transglutaminase 2. Average masses were estimated using a standard Gaussian fitting over the respective peaks. For each measurement, an average of 3000 counts was targeted as a quality control measure to produce comparable results, and the sample concentrations were adjusted accordingly.

### 2.16 Statistical Analysis

Individual data points, mean, standard deviation, and confidence intervals (CIs) are displayed in the figures. The used statistical tests are indicated in the figure legends. Visualization and statistical analysis were performed with GraphPad Prism 7 and R (version 4.3.2.) using the following packages: Dabestr 0.3.0, Pacman 0.5.1, rio 1.0.1, tidyverse 2.0.0, ggrepel 0.9.5, RColorBrewer 1.1‐3, svglite 2.1.3, ggpubr 0.6.0, rstatix 0.7.2, effsize 0.8.1. Data were tested for normality using the Shapiro–Wilk test with a violation limit of *p* < 0.01. Datasets that were not normally distributed were analyzed using nonparametric tests. Datasets that were normally distributed were analyzed with the indicated parametric tests. No test for outliers was conducted, and no data points were excluded from the analyses. Statistical significance is designated as *p < 0.05, **p < 0.01, ***p < 0.001, and ****p < 0.0001. For each experiment, the number of cells (*n*) and biological/technical replicates (*n*) are reported within the figure legends.

### 2.17 Ethics Statement

All relevant ethical regulations for animal testing and research were followed, and the experiments were authorized by the local research ethics committees (Germany, Landesamt für Natur, Umwelt und Verbraucherschutz Nordrhein‐Westfalen, reference 2021.A450).

## 3 Results

### 3.1 Gephyrin‐CB2_SH3_ _‐_ Complex Formation Requires Gephyrin Oligomerization

Gephyrin E‐domain dimerization is essential for synaptic gephyrin clustering (Saiyed et al. 2007). However, within full‐length gephyrin, E‐domain dimerization is inhibited by the C‐domain (Bedet et al. 2006; Sander et al. 2013). We probed whether binding events within the gephyrin C‐domain, such as the interaction with CB (Harvey et al. 2004), could relieve this inhibitory effect and thereby induce gephyrin oligomerization. To study the oligomerization of gephyrin upon CB binding, size exclusion chromatography (SEC) based interaction studies, using recombinantly expressed and purified proteins, were performed (Figure 1). SEC, dynamic light scattering, chemical cross‐linking, and atomic force microscopy studies revealed that recombinant full‐length gephyrin expressed in *E. coli* forms stable trimers alongside higher oligomeric states (Saiyed et al. 2007; Sander et al. 2013; Schrader et al. 2004; Sola et al. 2004). Therefore, the different oligomeric states of *E. coli* expressed and affinity‐purified gephyrin were separated via SEC (Figure S1A), resulting in gephyrin trimers (GephT, 1.83 mL = 315 kDa, Figure 1C) and gephyrin high oligomers (GephHO, 1.50 mL = 1234 kDa, Figure 1D). To study the complex formation between the different oligomeric states of recombinant gephyrin (GephT and GephHO) and the constitutively active (open conformation) CB2 splice variant lacking the SH3 domain (CB2_SH3‐_, Figure 1B) (Harvey et al. 2004), equimolar amounts of both proteins were co‐incubated (30 min) and subsequently analyzed by SEC (Figure 1C,D). Surprisingly, in the case of GephT, no complex formation with CB2_SH3‐_ was observed (Figure 1C). In contrast, GephHO formed a high‐molecular weight complex with CB2_SH3‐_, eluting at the void volume of the SEC column, corresponding to a molecular weight (MW) larger than 5 MDa on the basis of the fractionation limit of the resin (Figure 1D).

Blue‐native PAGE analysis of gephyrin from pig brain revealed the presence of cytosolic gephyrin trimers and membrane‐associated higher oligomers (Figure S1B). The observed band height of cytosolic gephyrin from brain extracts corresponded to the size of recombinant GephT, whereas the band height of membrane‐associated gephyrin corresponded to the size of recombinant GephHO (Figure S1B). Thus, our data suggest that trimeric gephyrin is mainly found within the cytosol, whereas the formation of distinct larger gephyrin oligomers is induced at submembranous sites. In conclusion, gephyrin‐CB2_SH3‐_ complex formation depends on the oligomeric state of gephyrin, as higher gephyrin oligomers, found at submembranous sites, preferentially engage in complex formation with CB2_SH3‐_ compared to trimeric gephyrin, which is mainly found within the cytosol.

### 3.2 Lipids Stabilize the Gephyrin‐CB2_SH3_ _‐_ Complex

The ability of CB to bind PIPs is crucial for gephyrin trafficking towards the synaptic membrane (Harvey et al. 2004; Papadopoulos et al. 2017). To test the effect of lipids on the high‐molecular weight gephyrin‐CB2_SH3‐_ complex formation, we performed similar SEC experiments in the presence of bovine brain lipid extract Folch fraction I (from here on referred to as Folch) (Eggers and Schwudke 2016).

For the SEC studies, Folch lipids were solubilized in DDM detergent micelles and added to equimolar amounts of CB2_SH3‐_ and the respective gephyrin multimers (Figure 1E,F). The addition of DDM alone did not alter the gephyrin‐CB2_SH3‐_ complex formation in the case of both oligomeric states compared to the condition without any detergent (Figure S2E,F). As expected, CB2_SH3‐_ alone did interact with the Folch‐DDM micelles, as a peak shift was detected when compared to the conditions without Folch (Figure S2B). The elution profile of GephT and GephHO was not altered by the addition of Folch lipids, indicating that the lipids did not affect the oligomerization of gephyrin (Figure S2C,D).

The addition of Folch lipids promoted complex formation with CB2_SH3‐_ for both oligomeric states of gephyrin (Figure 1E,F). In the case of GephHO, the addition of Folch lipids induced a significant increase in the void volume peak (> 5 MDa, P1, Figure 1F) compared to the condition without lipids (Figure 1D,I, *p* = 0.0151). Surprisingly, in the presence of Folch lipids, GephT also formed a complex with CB2_SH3‐_, eluting at the void volume of the column (> 5 MDa, P1, Figure 1E), whereas in the absence of lipids, no peak shift was detected (Figure 1C,I, *p* = 0.0299). The height of the void volume peak P1 was significantly smaller in the case of GephT (Figure 1E) compared to the complex formed with GephHO (Figure 1F,I, *p* = 0.0004), indicating that also in the presence of Folch lipids, the gephyrin‐CB2_SH3‐_ complex formation is less efficient for GephT compared to GephHO. In summary, lipid binding to CB strengthened the interaction with gephyrin, enabling complex formation with both oligomeric states of gephyrin.

Previous studies mapping the binding sites of gephyrin and CB suggest that one gephyrin monomer binds to one CB2_SH3‐_ monomer (Grosskreutz et al. 2001; Harvey et al. 2004). Thus, GephT (315 kDa, Figure 1C) harbors three potential binding sites for CB2_SH3‐_ (63 kDa, Figure 1C–F), whereas GephHO (1234 kDa, Figure 1D), containing approximately four gephyrin trimers, harbors 12 potential binding sites. On the basis of the MWs determined by comparison to the elution of standard proteins (Figure S2A), the resulting complexes would correspond to a MW of approximately 504 kDa for GephT and 1990 kDa for GephHO. Thus, the formed > 5 MDa gephyrin‐CB2_SH3‐_ complex exceeds the theoretical MW of a 1:1 complex for GephT (~10‐fold) and for GephHO (~2.5‐fold). We therefore conclude that either a conformational change was induced upon CB2_SH3‐_ binding, leading to an extended hydrodynamic radius, or additional gephyrin and/or CB2_SH3‐_ subunits were associated to enlarge the gephyrin‐CB2_SH3‐_ complex.

### 3.3 High‐Molecular‐Weight Gephyrin‐CB2_SH3_ _‐_ Complex Is Based on Gephyrin Self‐Oligomerization via E‐Domain Dimerization

To address the question of whether the formation of the high‐molecular weight GephHO‐CB2_SH3‐_ complex is based on gephyrin oligomerization via E‐domain dimerization, we performed SEC interaction studies with oligomerization‐deficient gephyrin variants. Therefore, an E‐domain dimerization‐deficient gephyrin variant (ddGeph) was generated by introducing two amino acid exchanges in the E‐domain (G483D and S505F) both located outside the CB binding motif (Figure 1A). Krausze et al. (2017) identified specific mutations in the plant ortholog of gephyrin, Cnx1, disrupting the dimerization of the E‐domain without altering the overall structure of the protein (Krausze et al. 2017). Since single amino acid exchanges only partially shifted the equilibrium from dimerization to monomerization of Cnx1E (Krausze et al. 2017), we targeted both amino acid residues in gephyrin to achieve full E‐domain monomerization.

G483D is located within an α‐helix that is directly involved in the dimerization interface, whereas S505F is located within a structurally conserved β‐sheet in close proximity to an α‐helix within the dimerization interface (Figure S3A). To study the effect of the dual substitution on E‐domain dimerization, we characterized the oligomerization behavior of the isolated gephyrin E‐domain carrying both substitutions (ddGephE). SEC experiments revealed that ddGephE elutes with a smaller hydrodynamic radius than that of the WT‐gephyrin E‐domain (WT‐GephE, Figure S3C), being consistent with a switch from the dimeric to the monomeric state. Additionally, mass photometry measurements confirmed that the measured MW of ddGephE (49 ± 2.6 kDa) correlates to a monomer (theoretical MW = 47 kDa), whereas the measured MW of WT‐GephE (89 ± 1.7 kDa) matches that of a dimer (theoretical MW = 94 kDa, Figure S3D,E).

SEC studies of full‐length ddGeph directly after affinity purification revealed that besides trimer formation, no higher oligomeric states were observed (Figure S3B), indicating that E‐domain dimerization is essential for the formation of GephHO. As WT‐GephT formed a high‐molecular weight complex with CB2_SH3‐_ exclusively when Folch lipids were present (Figure 1C,E), we repeated those experiments in the presence of Folch lipids now with trimeric ddGeph (ddGephT). Interestingly, ddGephT did not form a complex together with CB2_SH3‐_ and Folch lipids (Figure 1G). This finding further supports our hypothesis that the high‐molecular weight gephyrin‐CB2_SH3‐_ complex is based on a gephyrin oligomerization that requires E‐domain dimerization (Figure 1J,K).

The formation of a gephyrin scaffold additionally requires the trimerization of the G‐domain (Saiyed et al. 2007). Therefore, we again performed SEC experiments with WT‐GephE, which still contains the entire CB binding motif but lacks the trimerizing G‐domain (Figure 1A). Again, no complex formation with CB2_SH3‐_ and Folch lipids was detected (Figure 1H). This finding led us to conclude that G‐domain trimerization is also required for the high‐molecular weight complex formation.

In summary, our results using oligomerization‐deficient gephyrin variants show that both gephyrin E‐domain dimerization as well as G‐domain trimerization are required for the high‐molecular weight gephyrin‐CB2_SH3‐_ complex formation. Thus, we conclude that the formation of the high‐molecular weight gephyrin‐CB2_SH3‐_ complex is based on gephyrin self‐oligomerization induced by interaction with CB2_SH3‐_ (Figure 1J,K).

### 3.4 Gephyrin‐CB2_SH3_ _‐_ Complexes Are Functionally Active

To confirm that the > 5 MDa gephyrin‐CB2_SH3‐_ complex contains properly folded, active proteins, we performed a functional analysis of the complex (Figure S4). Gephyrin catalyzes the last two steps of Moco biosynthesis, with the G‐domain catalyzing the adenylation of molybdopterin (MPT) and the E‐domain promoting the incorporation of molybdate into activated MPT‐AMP (Figure S4A) (Feng et al. 1998; Stallmeyer et al. 1999). We performed an in vitro assay (Belaidi and Schwarz 2013) to measure Moco production by GephHO in complex with CB2_SH3‐_. The resulting enzymatic activity was not impaired, as no significant difference in Moco production compared to GephHO alone was observed. Also, the addition of Folch lipids did not alter Moco synthesis of GephHO within the complex (Figure S4B).

Next, we investigated whether the neuronal receptor binding function of gephyrin is preserved in the > 5 MDa gephyrin‐CB2_SH3‐_ complex. The interaction between the inhibitory neurotransmitter receptors and gephyrin is facilitated through the intracellular cytosolic domains (ICDs) of the receptors (Kowalczyk et al. 2013; Meyer et al. 1995; Tretter et al. 2011). Therefore, we studied whether the gephyrin‐CB2_SH3‐_ complex can recruit binding peptides of inhibitory neurotransmitter receptors using a recently established pentameric soluble protein scaffold (Macha, Grünewald, et al. 2022). The scaffold is based on the insertion of the full‐length ICDs of the GlyR α‐ and β‐subunits into the soluble pentameric yeast lumazine synthase, resulting in the heteropentameric protein LS‐αβICD (Figure S4C) with an average subunit ratio of 2 α‐ and 3 β‐subunits. As the GlyR and GABA_A_R compete for the same binding pocket within the interface of the dimerized gephyrin E‐domain (Maric et al. 2014), the GlyR ICDs represent a suitable model to study the receptor binding ability.

We performed SEC experiments to investigate whether LS‐αβICD is recruited to the > 5 MDa GephHO‐Folch‐CB2_SH3‐_ complex (Figure S4D). Indeed, the peak corresponding to LS‐αβICD (P3, 176 kDa, dashed gray line) was reduced, whereas the dominant void volume peak (P1) increased, indicating that LS‐αβICD interacts with the > 5 MDa GephHO‐Folch‐CB2_SH3‐_ complex (P1, bold dark blue line, Figure S4D). In contrast, LS‐αβICD and GephHO alone only formed a smaller complex of 1397 kDa in size (P2, bold light blue line, Figure S4D). No interaction of LS‐αβICD with CB2_SH3‐_ was observed in our SEC studies (bold orange line, Figure S4D). The LS‐αβICD construct is derived from the rat GlyR α1‐ICD, which differs from the human/mouse sequence previously implicated in CB binding by one amino acid within the polyproline II (PPII) motif (Breitinger et al. 2020).

The LS‐αβICD‐GephHO‐CB2_SH3‐_ complex formation was further confirmed via SDS‐PAGE analysis of corresponding peak fractions (Figure S4E). The band ratio of LS‐αβICD within the void volume peak P1 was increased to 63% for the LS‐αβICD‐GephHO‐CB2_SH3‐_ complex compared to 20% and 11% in the case of LS‐αβICD with GephHO alone, respectively. These results further support the conclusion that LS‐αβICD was recruited by the > 5 MDa GephHO‐CB2_SH3‐_ complex. In summary, gephyrin within the > 5 MDa gephyrin‐CB2_SH3‐_ complex remained enzymatically active and is able to recruit inhibitory neurotransmitter receptor binding sites, which are essential for the proper function of gephyrin‐CB clusters at inhibitory postsynapses.

### 3.5 PIPs, Present at the Plasma Membrane, Stabilize the Gephyrin‐CB2_SH3_ _‐_ Complex

Next, we aimed to determine which types of lipids promote gephyrin‐CB2_SH3‐_ complex formation. CB is known to interact with PIPs via its PH domain with a high specificity for PI(3)P (Kalscheuer et al. 2009; Papadopoulos et al. 2017), whereas interactors like TC10 or NL‐2 induce a phospholipid affinity switch towards plasma‐membrane‐resident PIPs, such as PI(3,4)P_2_, PI(4,5)P_2_ and PI(3,4,5)P_3_ (Kilisch et al. 2020; Schäfer et al. 2020).

**FIGURE 2:**
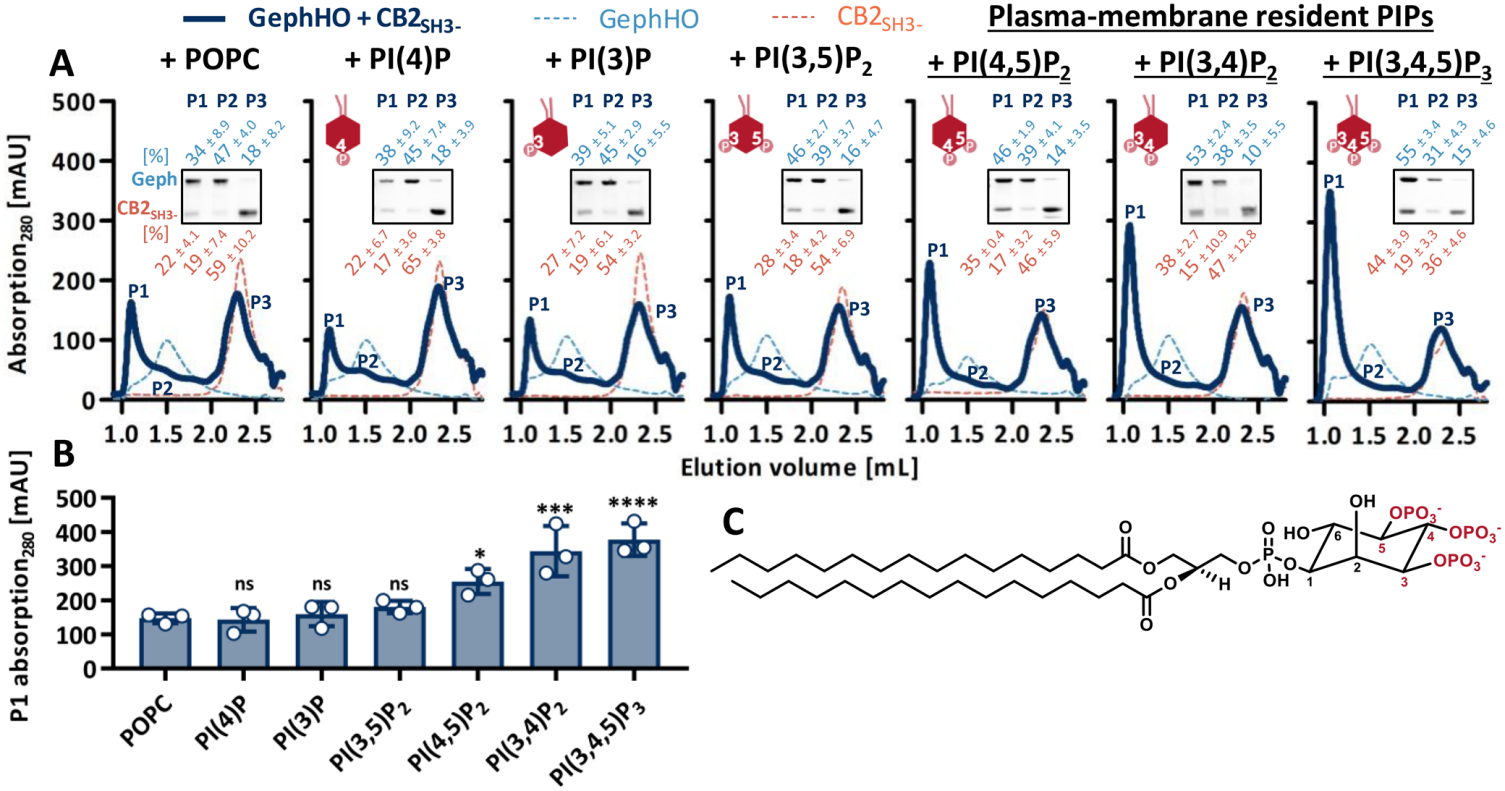
Plasma‐membrane‐resident PIPs stabilize the high‐molecular weight gephyrin‐CB2_SH3‐_ complex. (A) Representative SEC elution profiles of GephHO mixed with CB2_SH3‐_ at equimolar ratios, in the presence of the respective POPC‐/PIP‐lipid DDM micelles (dark blue line). Single GephHO (dashed line, light blue) and CB2_SH3‐_ (dashed line, orange) together with the respective POPC‐/PIP‐lipid DDM micelles serve as a reference within each graph. Insets depicting SDS‐PAGE analysis of peak 1 (P1), peak 2 (P2), and peak 3 (P3) of the respective GephHO‐CB2_SH3‐_ interaction run. Numbers above and below the representative SDS‐PAGE image represent the mean relative band intensity ± SD [%] of GephHO (light blue) and CB2_SH3‐_ (orange), respectively, between P1, P2, and P3 (*n* = 3 from three independently purified protein batches). Depiction of the used PIP created with BioRender.com. (B) Quantification of P1 absorption at 280 nm expressed as mean ± SD (*n* = 3 from three independently purified protein batches). PIP treated conditions were compared to the POPC control condition, revealing that P1 absorption is significantly increased for plasma‐membrane‐resident PIPs (1way ANOVA *F*(6,14) = 16.64, *p* < 0.0001, Dunnett's post hoc test: PI(4)P *p* = 0.9998 (ns); PI(3)P *p* = 0.9980 (ns); PI(3,5)P_2_ *p* = 0.8317 (ns); PI(4,5)P_2_ *p* = 0.0306 (*); PI(3,4)P_2_ *p* = 0.0003 (***); PI(3,4,5)P_3_ *p* = 0.0001 (****)). (C) Molecular structure of a PIP with the possible phosphorylation sites at the inositol ring highlighted in red (created with ChemDraw).

To study the effect of PIPs on the gephyrin‐CB2_SH3‐_ complex formation, we doped POPC DDM micelles with 10 mol% of different PIPs, namely PI(3)P, PI(4)P, PI(3,4)P_2_, PI(3,5)P_2_, PI(4,5)P_2_, and PI(3,4,5)P_3_ (Figure 2C). Our subsequent PIP ‘screen’ was performed with GephHO as it showed a more stable complex formation with CB2_SH3‐_ than GephT (Figure 1C–F). To study the effect of the PIPs, the respective lipid‐DDM micelles were added to equimolar amounts of CB2_SH3‐_ and GephHO, and complex formation was studied by SEC (Figure 2A). The addition of POPC (Figure 2A) did not alter the gephyrin‐CB2_SH3‐_ complex formation as compared to DDM only or no additive added (Figure S2F).

The phosphatidylinositol monophosphates PI(3)P and PI(4)P as well as PI(3,5)P_2_ did not improve the gephyrin‐CB2_SH3‐_ complex formation as the height of the > 5 MDa peak P1 did not increase compared to the POPC control (Figure 2A,B). Interestingly, only PIPs known to be present at the plasma membrane, PI(3,4)P_2_, PI(4,5)P_2_, and PI(3,4,5)P_3_ (Ueda 2014), did significantly increase the height of P1 compared to the POPC control condition (Figure 2A,B). The presence of phosphatidylinositol triphosphate PI(3,4,5)P_3_ induced the strongest increase of P1 with 378 ± 39 mAU (*p* < 0.0001), followed by PI(3,4)P_2_ with 344 ± 60 mAU (*p* = 0.0003) and PI(4,5)P_2_ with 255 ± 30 mAU (*p* = 0.0306) compared to 148 ± 12 mAU for POPC (Figure 2B).

Changes in P1 height were further confirmed by SDS‐PAGE analysis of the peak fractions, showing the respective increase of band intensity corresponding to gephyrin and CB2_SH3‐_ within the > 5 MDa complex compared to the control condition using POPC (insets Figure 2A). Again, the strongest increase was observed for PI(3,4,5)P_3_ with an increase of 21% GephHO and 22% CB2_SH3‐_ within P1 compared to the POPC control. In summary, our SEC studies showed that PIPs present at the plasma membrane promote high‐molecular weight gephyrin‐CB2_SH3‐_ complex formation, indicating that the gephyrin‐CB scaffold is stabilized and maintained at synaptic membranes via the interaction with those PIPs.

### 3.6 Phosphomimicking Mutant S325D‐Gephyrin Does Not Form a Stable Complex With CB2_SH3_ _‐_

**FIGURE 3:**
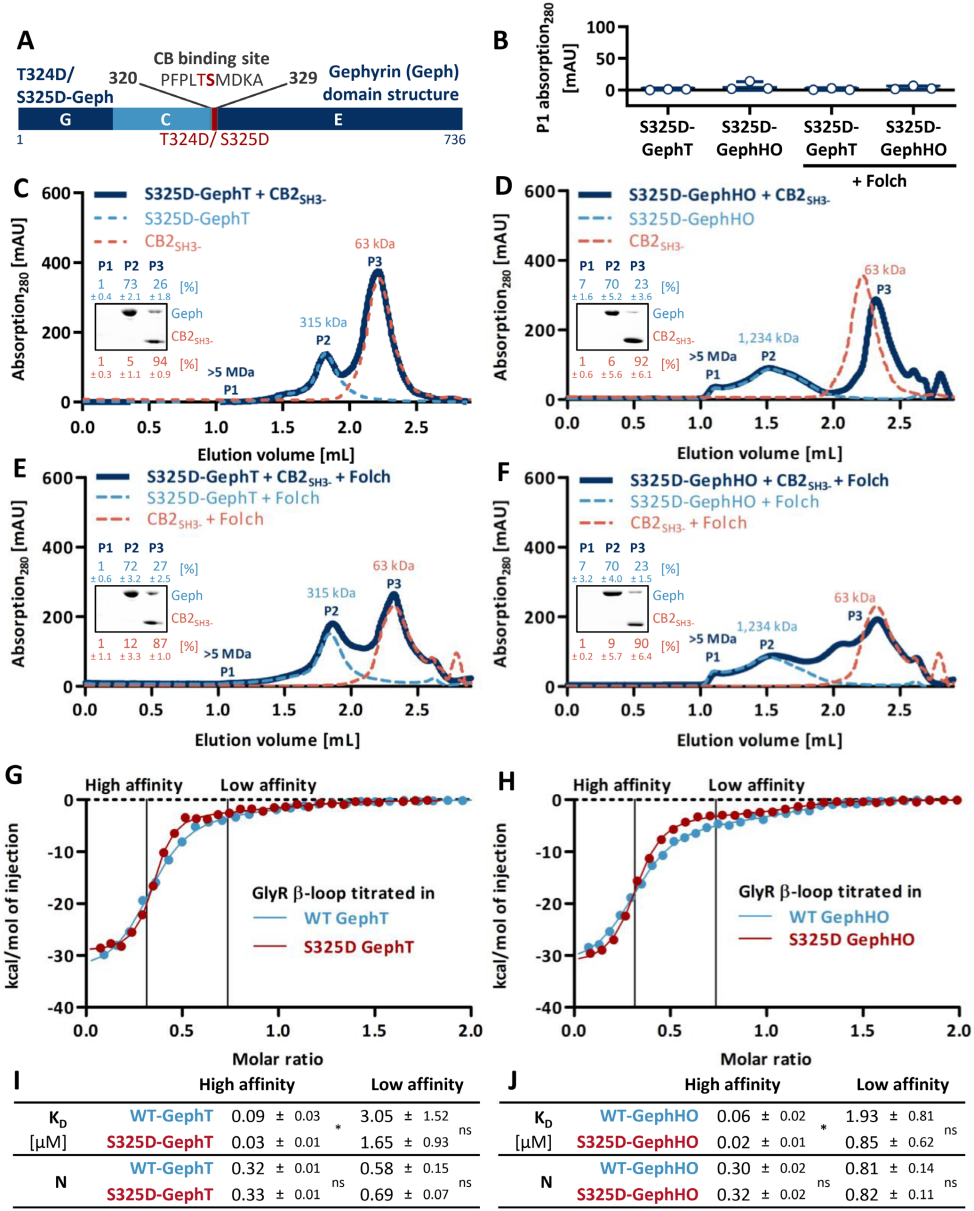
S325D‐Geph does not form a complex with CB2_SH3‐_, whereas the receptor binding ability is improved. (A) Domain structure of the phosphomimicking mutant T324D‐Geph and S325D‐Geph with the phosphorylation sites highlighted in red. (B–F) SEC interaction studies of the different oligomeric states of S325D‐Geph mixed with CB2_SH3‐_ at equimolar ratios. (B) Quantification of peak 1 (P1) absorption at 280 nm expressed as mean ± SD (*n* = 3 from three independently purified protein batches). One‐way ANOVA revealed no significant differences between conditions (*F*(3,8) = 1.379, *p* = 0.3175). (C–F) Representative SEC elution profiles of S325D‐Geph mixed with CB2_SH3‐_ (dark blue line), alone or in the presence of Folch. Single S325D‐Geph (dashed line, light blue) and CB2_SH3‐_ (dashed line, orange), with or without Folch, serve as a reference within each panel. The MWs of the single proteins, determined according to the standard protein calibration curve, are indicated. Insets depict SDS‐PAGE analysis of peak 1 (P1), peak 2 (P2), and peak 3 (P3) of the S325D‐Geph–CB2_SH3‐_ interaction run. Numbers above and below the representative SDS‐PAGE image represent the mean relative band intensity ± SD [%] of S325D‐Geph (light blue) and CB2_SH3‐_ (orange), respectively, between P1, P2, and P3 (*n* = 3 from three independently purified protein batches). (G, H) Representative ITC binding isotherms of the GlyR β‐loop titrated into the respective oligomeric state of WT‐Geph or S325D‐Geph. The binding data points were fitted using a two‐side model (line) with the high and low affinity binding sites highlighted. (I, J) Binding parameters derived from the ITC experiments, including *K*_D_ (μM) and N (molar ratio). Results are expressed as mean ± SD (*n* = 4 from three independently purified protein batches) and were analyzed using unpaired, two‐tailed Student's *t*‐test. For the high affinity binding site, the *K*_D_ value was significantly decreased for both oligomeric states of S325D‐Geph compared to WT‐Geph (GephT: *p* = 0.0113 (*); GephHO: *p* = 0.0110 (*)). All other parameters were not significantly altered (ns = *p* ≥ 0.05).

Gephyrin is subject to various PTMs such as phosphorylation, palmitoylation, and nitrosylation that can affect the structure and scaffolding properties of gephyrin, its trafficking, as well as its ability to interact with partner proteins (Tyagarajan, Ghosh, Yévenes, et al. 2011). There are two known phosphorylation sites within the CB binding motif of gephyrin, Thr324 and Ser325 (Herweg and Schwarz 2012; Ogino et al. 2019). We studied the impact of both phosphorylations on the gephyrin‐CB complex formation by exchanging the respective residues to aspartates, resulting in the phosphomimicking variants T324D‐Geph and S325D‐Geph (Figure 3A).

Both phosphomimicking variants showed WT‐like oligomerization behavior following recombinant expression and purification, indicating that gephyrin oligomerization is not affected by the amino acid exchanges (Figure S5A). Therefore, GephT and GephHO of the phosphomimicking variants were isolated and used for SEC interaction studies with CB2_SH3‐_. T324D did not alter the gephyrin‐CB2_SH3‐_ complex formation (Figure S5C–F) and was therefore not subjected to further experiments. In contrast, in the case of S325D‐Geph, no complex formation with CB2_SH3‐_ could be observed regardless of the oligomeric state used (Figure 3B–D). Also, the addition of Folch lipids could not recover the complex formation (Figure 3B,E,F). In conclusion, the phosphomimicking variant S325D within the CB binding site of gephyrin abolished the complex formation with CB2_SH3‐_, suggesting a negative impact of phosphorylation at Ser325 on CB binding.

### 3.7 S325D‐Geph Shows Enzymatic Activity and Increased GlyR β‐Loop Binding

To investigate whether the phosphomimicking variant S325D affects not only the complex formation with CB2_SH3‐_ but also other functions of gephyrin, we investigated Moco synthesis and receptor peptide binding. Performing the previously described in vitro Moco assay (Belaidi and Schwarz 2013) confirmed that the enzymatic activity of S325D‐Geph was not altered (Figure S5H).

The receptor binding ability of S325D‐Geph was investigated by ITC. Therefore, a peptide comprising the main binding motif of the GlyR β‐ICD (residues 378–426, GlyR β‐loop) was titrated into the trimeric and high oligomeric state of S325D‐Geph and WT‐gephyrin (WT‐Geph; Figure 3G–J and Figure S6). Two binding sites were described for the interaction between gephyrin and the GlyR β‐loop, displaying a high‐affinity binding site in the sub‐micromolar range and a low‐affinity binding site with micromolar affinity (Grünewald et al. 2018; Herweg and Schwarz 2012). Our ITC measurements revealed that both oligomeric states of S325D‐Geph were able to interact with the GlyR β‐loop, resulting in exothermic binding events, revealing the low‐ and high‐affinity binding site (Figure 3G–J). The stoichiometry of the formed complexes was comparable to WT‐Geph in the case of both binding sites (Figure 3I,J). Also, the thermodynamic parameters of the interaction (binding enthalpy, entropy and free Gibbs energy) were not altered between S325D‐Geph and WT‐Geph (Figure S6E,F). However, in the case of the high‐affinity binding site, we observed a significantly increased, approximately three‐fold higher, affinity of S325D‐Geph compared to WT‐Geph (Figure 3I,J, GephT: *p* = 0.0113, GephHO *p* = 0.0110). Thus, the ITC measurements show that the phosphomimicking variant S325D exhibits an improved receptor binding.

### 3.8 S325D‐Geph Does Not Form Membrane‐Associated Microclusters Upon Co‐Expression With CB2_SH3_ _‐_ In HEK GPHN ^−/−^ Cells

**FIGURE 4:**
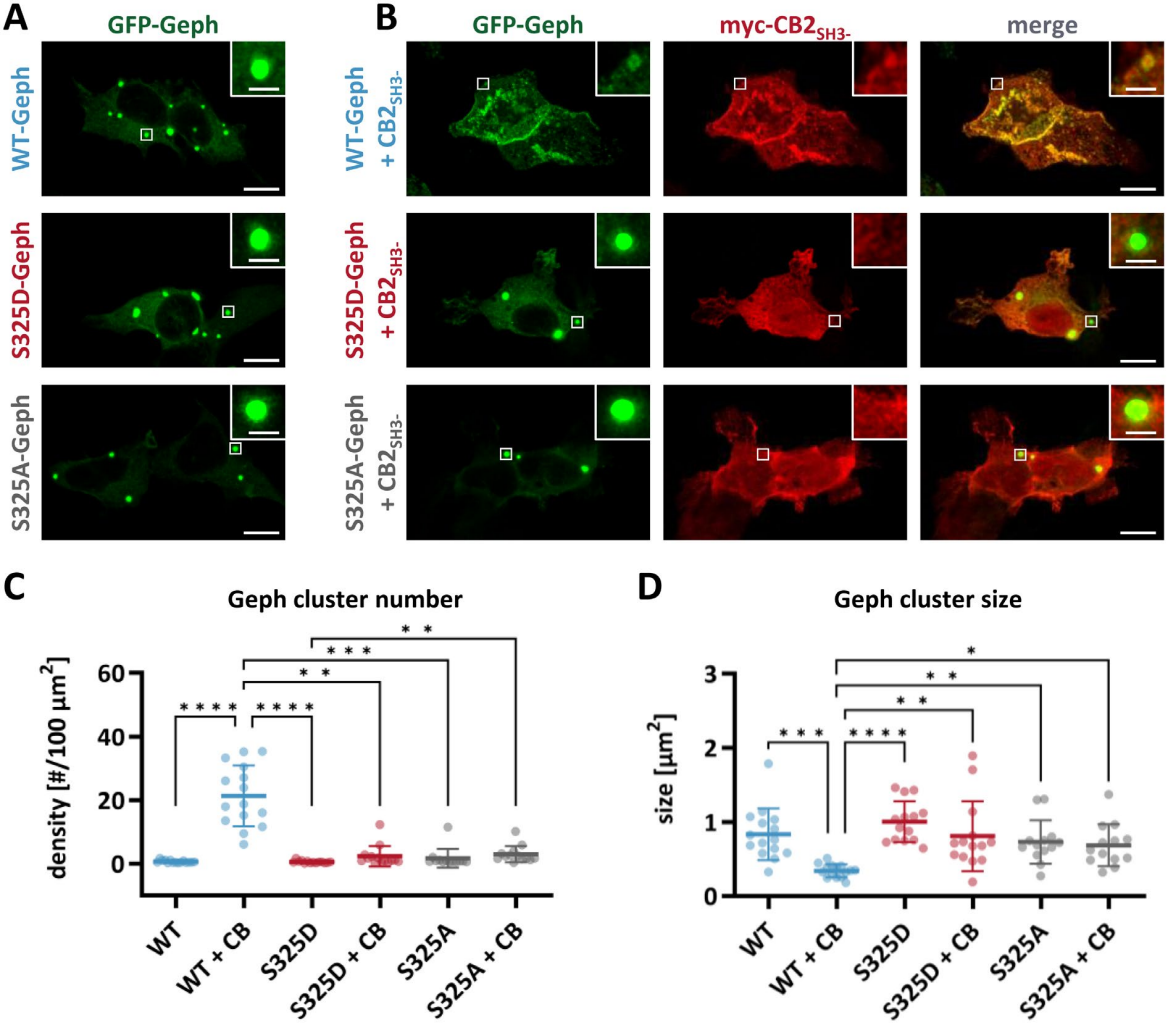
S325D‐ and S325A‐Geph do not form submembranous microclusters upon CB2_SH3‐_ co‐expression in HEK GPHN^−/−^ cells. (A) Representative confocal images of HEK GPHN^−/−^ cells expressing GFP‐tagged WT‐Geph, S325D‐Geph, or S325A‐Geph alone (B) or together with myc‐tagged CB2_SH3‐_ (scale bars = 10 μm). Insets show zoomed‐in views of protein clusters, with white boxes indicating the regions displayed (scale bars = 2 μm). CB2_SH3‐_ induces the formation of WT‐Geph microclusters at the plasma membrane. This microcluster formation is absent in the case of S325D‐ and S325A‐Geph. (C, D) Quantitative analysis of GFP‐Geph clusters with individual data points and mean ± SEM displayed in the figures (*n* = 13–15 images from three independent transfections). (C) Quantification of the GFP‐tagged Geph cluster number per 100 μm^2^ analyzed with a Kruskal–Wallis followed by Dunn's post hoc test (total: *H* = 53.67; WT vs. WT + CB: *p* < 0.0001 (****); WT vs. S325A + CB: *p* = 0.0137 (*); WT + CB vs. S325D: *p* < 0.0001 (****); WT + CB vs. S325D + CB: *p* = 0.0030 (**); WT + CB vs. S325A: *p* = 0.0002 (***); S325D vs. S325A + CB: *p* = 0.0040 (**)); all other comparisons were not significant (ns = *p* ≥ 0.05). (D) Quantification of the mean GFP‐Geph cluster size per image analyzed with a Kruskal–Wallis followed by Dunn's post hoc test (total: *H* = 37.12; WT vs. WT + CB: *p* < 0.0001 (****); WT + CB vs. S325D: *p* < 0.0001 (****); WT + CB vs. S325D + CB: *p* = 0.0038 (**); WT + CB vs. S325A: *p* = 0.0070 (**); WT + CB vs. S325A + CB: *p* = 0.0286 (*)); all other comparisons were not significant (ns = *p* ≥ 0.05).

Previous studies have demonstrated that co‐expression of gephyrin together with CB, in its open conformation, relocates gephyrin from large intracellular aggregates (“blobs”) into small microclusters (diameter, 0.2–0.5 μm) located at the plasma membrane in human embryonic kidney (HEK) 293 cells (Kins et al. 2000). Given that S325D‐Geph was not able to form a complex with CB2_SH3‐_ within our SEC studies (Figure 3B–F), we investigated microcluster formation of S325D‐Geph following co‐expression with CB2_SH3‐_ in HEK293 cells (Figure 4). Additionally, to assess the role of Ser325 in its non‐phosphorylated state, we included the S325A‐gephyrin variant (S325A‐Geph), lacking the hydroxyl group required for phosphorylation and potential hydrogen bonding. To rule out that endogenous gephyrin influences the clustering behavior of S325D‐Geph, the interaction studies were performed in gephyrin knock‐out HEK293 cells (HEK GPHN^−/−^, Figure S5G). Cells were transfected with GFP‐tagged gephyrin (GFP‐Geph) constructs (containing WT‐Geph, S325D‐Geph, or S325A‐Geph) either alone (Figure 4A) or together with myc‐tagged CB2_SH3‐_ (Figure 4B). When expressed alone, all GFP‐Geph variants formed large intracellular aggregates (Figure 4A) with no significant difference in the number or size of clusters between the respective gephyrin variants (Figure 4C,D). As expected, co‐expression with CB2_SH3‐_ resulted in redistribution of WT‐Geph into submembranous microclusters that co‐localized with CB2_SH3‐_ (Figure 4B), characterized by a significantly increased cluster number (*p* < 0.0001, Figure 4C), whereas the cluster size was significantly decreased (*p* < 0.0001, Figure 4D). In contrast, both S325D‐Geph and S325A‐Geph failed to redistribute into microclusters upon CB2_SH3‐_ co‐expression (Figure 4B), with no significant change in cluster number and size (Figure 4C,D). Additionally, no co‐localization with CB2_SH3‐_ was observed, which remained diffusely distributed in the cytoplasm (Figure 4B).

These findings indicate that the hydroxyl group of gephyrin Ser325 is essential for the interaction between gephyrin and CB2_SH3‐_, likely through hydrogen bonding. Substitution with either alanine or aspartate disrupts this interaction and thereby abolishes CB‐mediated submembranous microcluster formation. Importantly, phosphorylation of Ser325 would similarly impair hydrogen bonding by removing the hydroxyl hydrogen, suggesting a potential regulatory mechanism for gephyrin‐CB complex formation.

### 3.9 Impaired S325D‐Geph Clustering at GABAergic Synapses in Hippocampal Neurons

**FIGURE 5:**
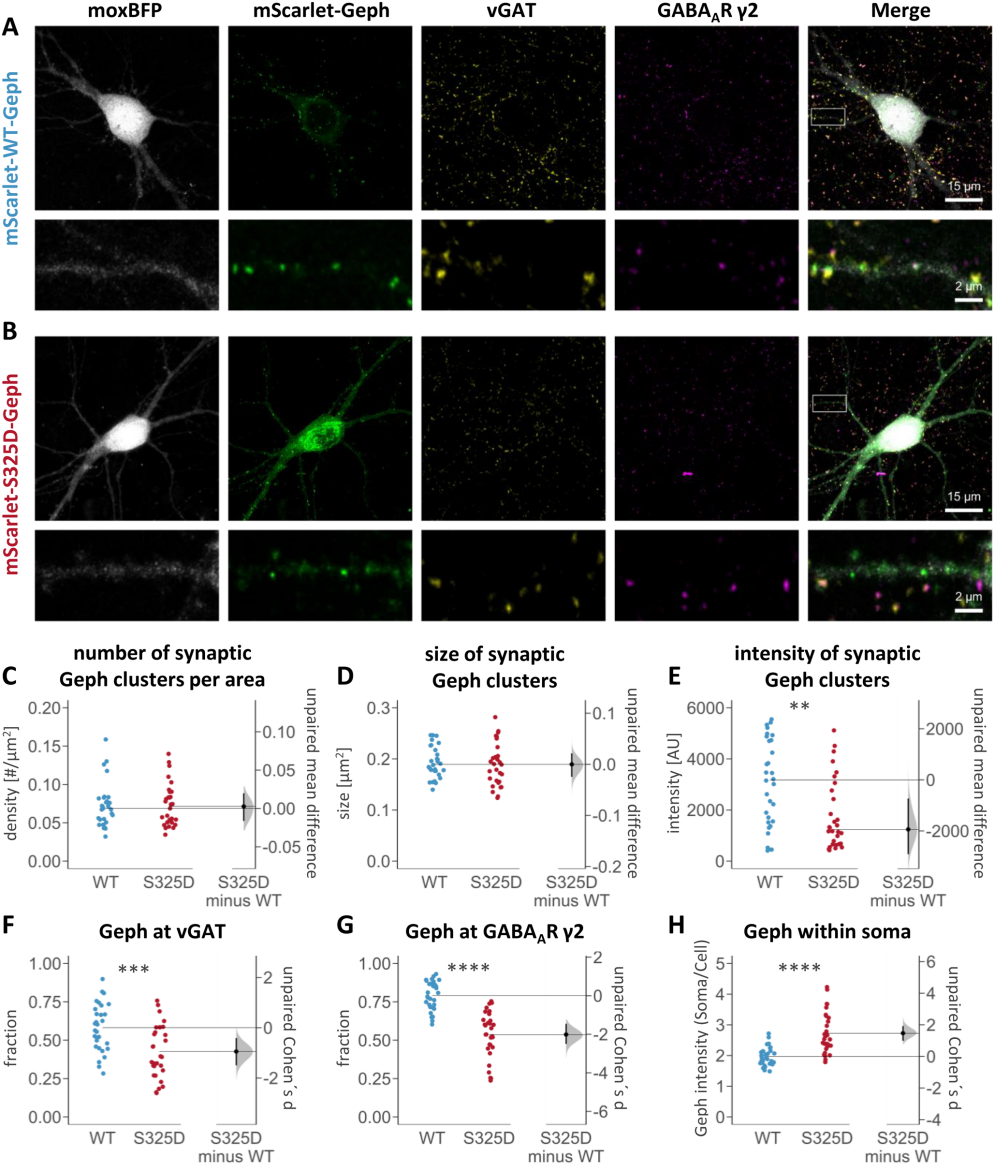
Impaired S325D‐Geph clustering at GABAergic synapses in murine hippocampal neurons. Representative confocal images of hippocampal neurons expressing moxBFP‐IRES‐Flpo and mScarlet‐tagged (A) WT‐Geph or (B) S325D‐Geph. (C–H) Quantitative analysis of mScarlet‐tagged Geph clusters was performed using an automated image analysis with the individual data points, mean, confidence intervals, and standard deviation displayed in the figures (*n* = 30 cells per condition from four independent cultures). (C, D) Average number (per μm^2^) as well as average size (per cell) of synaptic clusters (vGAT‐positive and GABA_A_Rɣ2‐positive) is not different between both variants; Wilcoxon rank test *p* = 0.786 (ns) and *p* = 0.542 (ns), respectively. (E) The average fluorescence intensity of synaptic S325D‐Geph clusters (per cell) is significantly reduced compared to WT‐Geph; Wilcoxon rank test *p* = 0.002 (**). (F, G) The fractions of vGAT‐ and GABA_A_Rɣ2‐positive clusters are significantly reduced in case of S325D‐Geph; Student's *t* test *p* = 0.0005 (***) and *p* = 4.1 × 10^−10^ (****), respectively. (H) Ratio of somatic/whole cell mean mScarlet‐Geph fluorescence intensity is significantly increased for S325D‐Geph compared to WT‐Geph; Student's *t* test *p* = 1.2 × 10^−6^ (****).

Numerous studies have demonstrated the importance of CB in the formation and maintenance of gephyrin clusters at GABAergic synapses in selected regions of the mammalian forebrain, including the hippocampus (Papadopoulos et al. 2007, 2008; Tyagarajan, Ghosh, Harvey, and Fritschy 2011). The phosphomimicking mutation S325D in gephyrin abolished complex formation with CB2_SH3‐_ both in vitro (Figure 3B–F) and in HEK GPHN^−/−^ cells (Figure 4). Therefore, clustering of S325D‐Geph was studied in murine hippocampal neurons using recombinant adeno‐associated virus (rAAVs) based expression (Figure 5) (Liebsch et al. 2023). CamKII‐positive glutamatergic cells were transfected with the recombinase Flpo and moxBFP as a cell filler. Flp‐dependent mScarlet‐WT‐Geph and mScarlet‐S325D‐Geph constructs were expressed using rAAVs. The synaptic localization of gephyrin clusters was quantified using immunocytochemistry of the GABA_A_R ɣ2‐subunit, as a marker of postsynaptic GABA_A_Rs, and the vesicular GABA transporter (vGAT), as a presynaptic marker (Figure 5C–H).

WT‐Geph and S325D‐Geph formed clusters throughout dendrites and regularly co‐localized with vGAT as well as the GABA_A_R ɣ2‐subunit (Figure 5A,B). Density (Figure 5C) and size (Figure 5D) of synaptically localized S325D‐Geph clusters were not altered. However, the intensity of synaptic S325D‐Geph clusters was significantly reduced compared to WT‐Geph (*p* = 0.002, Figure 5E). Furthermore, S325D‐Geph displayed a significantly reduced proportion of synaptically localized clusters, with only 42.63% ± 17.22% co‐localized with vGAT compared to 58.12% ± 15.47% for WT‐Geph (*p* = 0.0005, Cohen's *d* effect size *d* = −0.946, Figure 5F). The most drastic change, with the largest effect size, was observed in the proportion of gephyrin clusters co‐localized with the GABA_A_R γ2 subunit, which was significantly reduced to 53.70% ± 15.06% compared to 78.99% ± 9.43% for WT‐Geph (*p* ≤ 0.0001, Cohen's d effect size *d* = −2.012, Figure 5G). These results demonstrate that clustering of S325D‐Geph is impaired specifically at GABAergic synapses. Notably, clustering of the recombinantly expressed gephyrin variants was studied in the presence of endogenous gephyrin expressed in the neuronal cultures, suggesting that synaptic S325D‐Geph clusters potentially arise because of oligomerization with endogenous gephyrin. Additionally, the somatic localization of S325D‐Geph was significantly increased as the ratio of gephyrin intensity within the soma and the whole cell was increased to 2.73 ± 0.66 compared to 1.97 ± 0.30 for WT‐Geph (*p* ≤ 0.0001, Cohen's *d* effect size *d* = 1.470, Figure 5H).

Collectively, our cellular findings revealed a reduced association of S325D‐Geph with GABAergic synapses along with an increased somatic localization within hippocampal neurons. This indicates that the abolished complex formation of S325D‐Geph with CB, shown by SEC (Figure 3B–F) and HEK‐293 cell studies (Figure 4), inhibits the recruitment and clustering of S325D‐Geph at GABAergic synapses (Figure 5). These results highlight the importance of the high‐molecular weight gephyrin‐CB complex formation for proper GABAergic clustering and suggest a possible regulatory mechanism of gephyrin clustering at GABAergic synapses via phosphorylation at gephyrin Ser325.

## 4 Discussion

CB‐induced formation of gephyrin clusters at GABAergic synapses is of fundamental importance. However, the molecular mechanisms underlying the gephyrin‐CB complex formation remain poorly understood. On the basis of in vitro interaction and cellular studies using hippocampal neurons, we propose a model for the CB‐dependent gephyrin clustering involving gephyrin oligomerization induced by CB binding. This process is facilitated by the interaction of CB with plasma‐membrane‐resident PIPs and impaired by gephyrin Ser325 phosphorylation.

We discovered the formation of a high‐molecular weight gephyrin‐CB2_SH3‐_ complex, implying that the complex formation is based on gephyrin self‐oligomerization. Furthermore, variants of gephyrin, unable to form E‐domain dimers (ddGeph), or lacking the trimerizing G‐domain of gephyrin (GephE), were not able to form a high‐molecular weight complex together with CB2_SH3‐_. These results led us to conclude that CB2_SH3‐_ binding stabilizes the dimerized gephyrin E‐domain and thereby, together with G‐domain trimers, induces the formation of a gephyrin scaffold, resulting in the high‐molecular weight gephyrin‐CB2_SH3‐_ complex (Figure 1J,K).

A recent study, determining the affinity of the gephyrin‐CB interaction using FRET measurements, revealed that a monomeric (dimerization‐deficient) gephyrin E‐domain variant displayed a much lower affinity (*K*_D_ = 44.1 μM) toward CB2_SH3+_ compared to the dimerized E‐domain (*K*_D_ = 6.3 μM) (Imam et al. 2022). Their finding that E‐domain dimerization is not required for the initial binding event with CB but does enhance the affinity is in line with our conclusion that CB binding induces dimerization of the E‐domain within full‐length gephyrin. Potentially, CB initially binds to the domain boundary of the C‐domain and the monomerized E‐domain, relieving the auto‐inhibitory effect and thereby inducing E‐domain dimerization, which in turn increases the affinity of the gephyrin‐CB interaction.

An earlier study revealed that co‐expression of gephyrin and CB2_SH3‐_ in hippocampal neurons results in the formation of enlarged postsynaptic gephyrin clusters (0.81 ± 0.03 μm^2^), so‐called “superclusters”, compared to gephyrin clusters formed without CB co‐expression (0.24 ± 0.02 μm^2^) (Chiou et al. 2011). We propose that these “superclusters” are formed because of gephyrin network formation induced by CB2_SH3‐_ and stabilized by PIPs at the postsynaptic membrane. Gephyrin network formation on the basis of domain self‐oligomerization has been proposed for decades and was shown to be essential for gephyrin clustering in neuronal cells (Saiyed et al. 2007). Here, we provide direct in vitro evidence for a CB‐ and PIP‐dependent formation of such a gephyrin network.

Recent studies revealed that gephyrin network formation drives the formation of phase‐separated condensates, which is thought to be essential for compartmentalization of the inhibitory postsynaptic protein machinery into postsynaptic densities (Bai et al. 2021; Lee et al. 2024; Zhu et al. 2024). Thus, our finding that CB induces gephyrin network formation stabilized by PIPs at the plasma‐membrane potentially represents a molecular mechanism driving gephyrin condensate formation specifically at the postsynaptic membrane.

Recombinant gephyrin forms trimers (GephT) as well as distinct higher oligomeric states (GephHO). Our interaction studies revealed that the gephyrin‐CB complex formation is dependent on the oligomeric state of gephyrin. Although GephHO formed a high‐molecular weight complex with CB2_SH3‐_ alone, GephT only showed complex formation with CB2_SH3‐_ in the presence of lipids. The mechanism and structural basis for GephHO formation are still unclear. However, we showed that E‐domain dimerization is essential for the formation of GephHO as ddGeph, is unable to form E‐domain dimers, and did not form any higher oligomers. Studies showing that the presence of the C‐domain inhibits E‐domain dimerization were solely performed with isolated domains or full‐length GephT but not with GephHO (Bedet et al. 2006; Sander et al. 2013). Thus, it is possible that E‐domain dimerization within GephHO is favored and therefore the affinity for CB is increased compared to GephT with monomerized E‐domains.

An impaired formation of higher oligomers, as seen in the pathogenic G375D‐gephyrin variant, hampers clustering at GABAergic synapses and leads to epileptic encephalopathy, highlighting the importance of the formation of higher oligomers for synaptic gephyrin clustering (Kim et al. 2021). Indeed, for G375D‐gephyrin, CB‐induced submembranous microcluster formation in non‐neuronal cells was reduced (Dejanovic et al. 2015), which further supports our finding that the formation of higher gephyrin oligomers promotes the interaction with CB.

Our studies investigating the oligomeric state of native gephyrin from pig brain tissues indicate that cytosolic gephyrin is mainly trimeric, whereas membrane‐associated gephyrin is rather in a high‐oligomeric state. Therefore, we conclude that high‐molecular weight complex formation with CB is preferentially formed by higher oligomeric gephyrin at submembranous sites. On the other hand, intracellularly, gephyrin trimers with monomerized E‐domains bind CB with low affinity and require additional plasma membrane‐associated PIP interactions to induce gephyrin network formation (Figure 1J,K).

The ability of CB to bind PIPs is crucial for gephyrin targeting and clustering at CB‐dependent GABAergic synapses (Harvey et al. 2004; Papadopoulos et al. 2017). Our results demonstrate that PIPs not only act as a membrane tethering component but also stabilize and maintain the gephyrin‐CB complex at postsynaptic sites. Our finding that PIPs present at the plasma membrane, namely, PI(3,4)P_2_, PI(4,5)P_2_, and PI(3,4,5)P_3_ (Ueda 2014) promote the gephyrin‐CB complex formation is in line with the fact that those lipids are involved in the formation and maintenance of synapses (Dickson 2019; Ueda 2014; Volpatti et al. 2019). Consistently, PI(4,5)P_2_ was found to be required for CB‐dependent gephyrin microcluster formation within non‐neuronal cells (Kilisch et al. 2020). Within a cryo‐EM structure of a heteropentameric GABA_A_R, PI(4,5)P_2_ was identified to be bound to the cytosolic part of the α‐subunits (Laverty et al. 2018). PI(4,5)P_2_ binding did not alter the channel function and thus was thought to modulate its synaptic localization (Laverty et al. 2018; Mennerick et al. 2014). Interestingly, residues mediating PI(4,5)P_2_ binding are only conserved in synaptic α‐subunits (α1–3 and α5) and not in extrasynaptic subunits (α4 and α6) (Kasaragod and Schindelin 2019). Collectively, we conclude that GABA_A_R‐bound PI(4,5)P_2_ may recruit and stabilize the gephyrin‐CB complex at synaptic sites, thus facilitating the maintenance of gephyrin‐CB‐GABA_A_R clusters.

Besides the stabilization of gephyrin‐CB clusters at postsynaptic membranes via PIPs, we also identified a regulatory mechanism impairing CB‐induced gephyrin clustering via phosphorylation of gephyrin Ser325 within the CB binding motif. CaMKIIα‐dependent phosphorylation of gephyrin Ser325 was previously identified in zebrafish mauthner cells, where it enhances GlyR clustering by increasing the affinity between gephyrin and the GlyR β‐subunit (Ogino et al. 2019). Since gephyrin and GlyRs cluster independently of CB at glycinergic synapses (Papadopoulos et al. 2007, 2008), phosphorylation‐induced disruption of the gephyrin‐CB interaction is not expected to affect GlyR clustering. Given the critical role of glycinergic inhibition in mediating rapid escape responses in zebrafish, Ogino et al. (2019) focused specifically on the impact of Ser325 phosphorylation on GlyR clustering. Thus, the effect on CB‐dependent receptor clustering at GABAergic synapses remained elusive.

Our study shows that in hippocampal neurons, where CB is required for synaptic gephyrin clustering (Papadopoulos et al. 2007, 2008), the inability of the phosphomimicking variant S325D‐Geph to form a complex with CB caused an impairment in GABA_A_ receptor clustering. The increased somatic localization of S325D‐Geph is in line with a previous study, showing that gephyrin variants lacking the CB‐binding motif were located within somatic aggregates upon expression in cortical neurons (Harvey et al. 2004). The impaired complex formation between CB and S325D‐Geph potentially hampers CB‐dependent transport via PI(3)P‐containing sorting endosomes toward GABAergic synaptic sites, leading to an increased somatic localization (Papadopoulos et al. 2017). These results underscore the importance of the high‐molecular weight gephyrin‐CB complex for GABAergic synapse assembly. In aggregate, our findings suggest that Ser325 phosphorylation functions as a regulatory switch controlling synapse‐specific inhibitory scaffolding: it promotes GlyR clustering at CB‐independent glycinergic synapses because of an increased receptor affinity (Ogino et al. 2019) but impairs gephyrin clustering at CB‐dependent GABAergic synapses by disrupting gephyrin‐CB complex formation.

**FIGURE 6:**
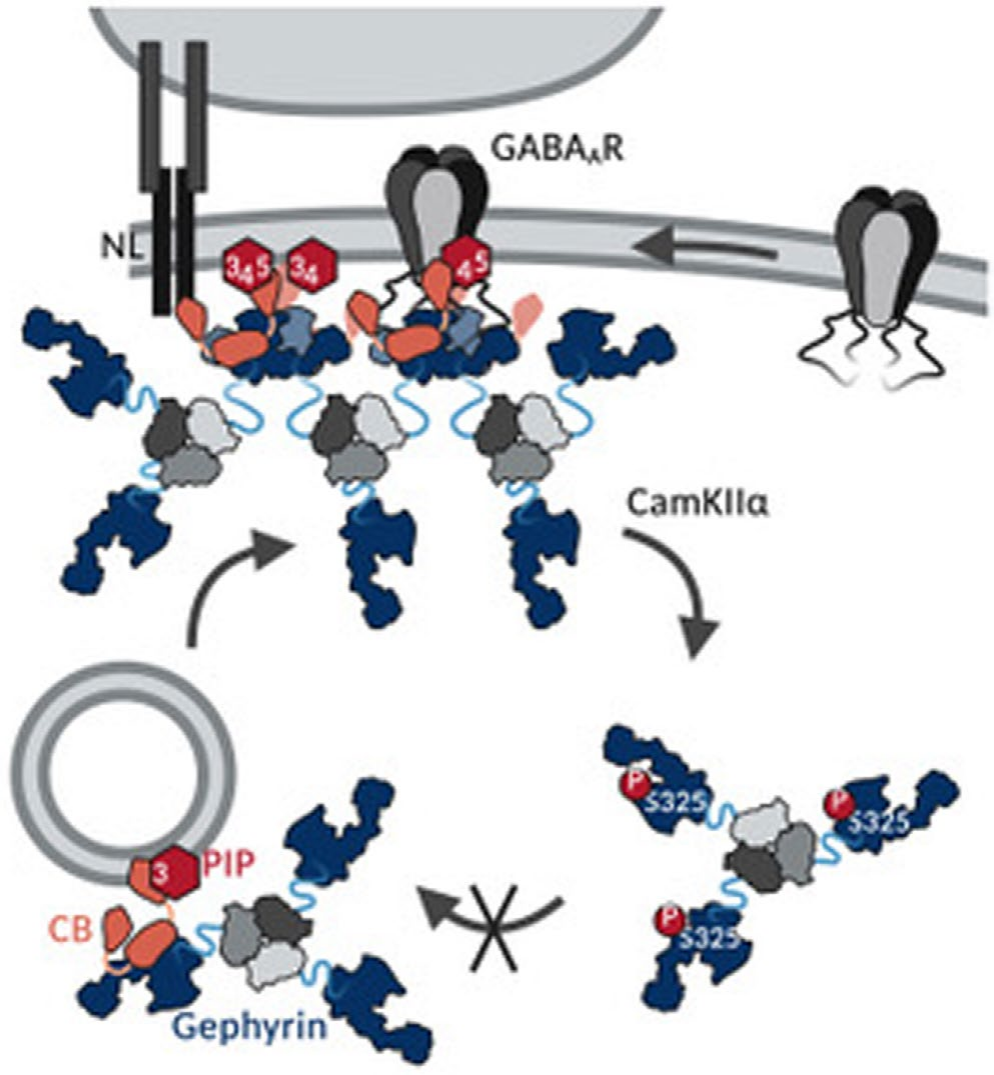
Proposed mechanism of gephyrin self‐oligomerization induced by CB at PIP‐containing postsynaptic membranes regulated via gephyrin phosphorylation. (A) Intracellularly, gephyrin trimers (GephT) with monomerized E‐domains bind to CB with a low affinity. Either gephyrin itself (Imam et al. 2022) or other interactors such as TC10 (Kilisch et al. 2020) activate CB, so that it specifically binds PI(3)P at early/sorting endosomes (Papadopoulos et al. 2017). Prolonged interaction with TC10 induces a phospholipid affinity switch of CB towards plasma‐membrane PIPs, resulting in the recruitment of the gephyrin‐CB complex towards the plasma membrane (Kilisch et al. 2020). (B) Interaction of CB with plasma‐membrane‐resident PIPs, PI(3,4,5)P_3_, PI(3,4)P_2_, and PI(4,5)P_2_, induces a conformational change within CB that promotes the interaction with the dimerized gephyrin E‐domain. Thereby, gephyrin E‐domain dimerization is stabilized, which leads to the formation of a postsynaptic gephyrin network. Additionally, because of an unknown mechanism, gephyrin associated with the plasma membrane adopts a higher oligomeric state (GephHO), which further promotes the complex formation with CB. PI(4,5)P_2_ bound to the cytosolic part of the GABA_A_R α‐subunits can additionally stabilize this gephyrin‐CB scaffold. (C) The CB and PIP induced gephyrin network can be regulated via phosphorylation of gephyrin Ser325 by CaMKIIα activation upon increased neuronal activity (Ogino et al. 2019). This phosphorylation impairs the complex formation with CB, thereby leading to the removal of gephyrin from gephyrin‐CB clusters at GABAergic synapses as well as an impaired transport via CB‐PI(3)P containing endosomes. Figure was created with BioRender.com.

Our in vitro interaction studies characterizing the gephyrin‐CB complex together with our studies in hippocampal neurons collectively propose a model for the CB‐ and PIP‐dependent gephyrin clustering at inhibitory postsynaptic sites (Figure 6). We suggest a CB‐induced gephyrin oligomerization via E‐domain dimerization that is stabilized at PIP‐containing postsynaptic plasma membranes of GABAergic synapses and downregulated via phosphorylation of gephyrin Ser325.

## Author Contributions

**Nele Burdina:** conceptualization, methodology, investigation, validation, visualization, writing – original draft, writing – review and editing. **Filip Liebsch:** methodology, writing – review and editing, supervision, investigation. **Arthur Macha:** methodology, writing – review and editing, supervision, investigation. **Joaquín Lucas Ortuño Gil:** methodology, investigation. **Pia Frommelt:** methodology, investigation. **Irina Rais:** methodology, investigation. **Fabian Basler:** methodology, investigation. **Simon Pöpsel:** methodology, writing – review and editing, investigation. **Guenter Schwarz:** conceptualization, writing – review and editing, supervision, project administration, funding acquisition.

## Conflicts of Interest

The authors declare no conflicts of interest.

## Peer Review

The peer review history for this article is available at https://www.webofscience.com/api/gateway/wos/peer‐review/10.1111/jnc.70169.

## Supporting information


Data S1

## Data Availability

Experimental raw data are available to be shared upon request to the corresponding authors. A preprint of this article was posted on bioRxiv; 21/01/2025; https://doi.org/10.1101/2025.01.20.633899.
